# Mechanical effects of sagittal variations on Pauwels type III femoral neck fractures treated with Femoral Neck System(FNS)

**DOI:** 10.1186/s12891-022-06016-y

**Published:** 2022-12-01

**Authors:** Chong Nan, Liang Ma, Yuechuang Liang, Yanjun Li, Zhanbei Ma

**Affiliations:** 1 Department of Orthopedic, Baoding No.1 Central Hospital, Baoding, 071000 Hebei Province China; 2 Baoding Digital Orthopaedic Key Laboratory, Baoding, 071000 Hebei Province China

**Keywords:** Femoral neck system, Femoral neck fractures, Finite element analysis, Biomechanics

## Abstract

**Background:**

The spatial position of internal fixation play a role in determining the stability of internal fixations, both in clinical practice and research. Researchers have examined the stability of FNS (Femoral neck system) in the presence of coronal plane changes. In our knowledge, due to the biomechanical limitations of the specimens, there are no mechanical studies on the sagittal variation of FNS. This study aimed to investigate the biomechanical behavior of sagittal variations on Pauwels type III femoral neck fractures treated with FNS through finite element analysis.

**Methods:**

Finite element models including Pauwels type III femoral neck fracture and FNS were reconstructed. Five fracture models(superior, central, inferior, anterior, posterior) were created in accordance with the bolt location in the sagittal plane within the femoral head. Equivalent stress, shear stress, and total deformation of each model under the same physiological load were recorded.

**Results:**

According to the results, the central model exhibited the slightest stress and displacement, with the exception of the superior model. The internal fixation stress of the superior model was smaller than that of the central model. However, the maximum interfragmentary stress, total deformation and shear resistance area of the superior model was larger than that of the central model.

**Conclusions:**

Central position of FNS in the sagittal plane allowed axial compression while reducing shear stress of internal fixation and interfragmentary equivalent stress. Off-axis fixation of the femoral neck increased the strain area and total displacement of the bone, raising the risk of fixation failure. Therefore, the central placement of FNS may be a better surgical target in the treatment of femoral neck fractures.

## Background

Internal fixation is the preferred treatment for young patients with Pauwels type III femoral neck fractures [1]. In light of the instability of these fractures, selecting and applying internal fixation remains a challenging task. The application of CSS (cannulated screws) and DHS (dynamic hip screw) to treat femoral neck fractures has become commonplace for many physicians [2]. As a result of the shear forces acting on unstable fractures (Pauwels type III), these strategies are prone to failure. Using a medial buttress plate, researchers have successfully optimized fixation techniques in order to resolve this issue [3, 4]. Although this procedure offers better biomechanical stability, some scholars have questioned its damage to blood vessels and soft tissues. With the recent approval of the FNS (Femoral Neck System), surgeons can achieve minimally invasive surgery while simultaneously reducing implant footprints [5–8].

Furthermore, an excellent biomechanics study has shown that the stability of FNS is comparable to DHS with an anti-rotation screw [9]. Many factors play a role in determining the stability of internal fixations, both in clinical practice and research. From a biomechanical point of view, the spatial location of the internal fixation has an effect on the overall stiffness of the model. Several articles have been published on the impact of primary nail position on the stability of internal fixation in intertrochanteric fractures, which is relevant to our study of FNS [10, 11]. In Kuzyk's study, he concluded that a lower placed lag screw produced the greatest axial and torsional stiffness [12]. While focusing on the proper tip-apex distance, we should also pay attention to the coronal and sagittal position variations. Researchers examined the stability of FNS in the presence of coronal plane changes [13]. In our knowledge, due to the biomechanical limitations of the specimens, there are no mechanical studies on the sagittal variation of FNS.

Accordingly, this study aimed to investigate the biomechanical behavior of sagittal variations on Pauwels type III femoral neck fractures treated with FNS through finite element analysis (FEA). In our research, we have been able to determine the peak values for displacement, shear stress, and equivalent stress (von Mises) by analyzing different finite element models.

## Material and methods

### Building proximal femur model

We recruited a 40-year-old female volunteer weighing 60 kg and measuring 160 cm who did not have hip or systemic diseases. Before the examination, written informed consent was obtained. A computed tomography scanner was used to scan the normal proximal femur of the volunteer. In Mimics 20.0 software, these images were imported as DICOM files. Following the predefined threshold, region growing, mask modification, and preliminary borders smoothing, the project was output in STL format. Using Geomagic—Wrap 2017 software, the model was further smoothed, the polygon mesh adjusted, and the surface fitted as well as imported into Solidworks 2017. Due to the irregularity of the proximal femur, more contour lines on the bony projections and borders were constructed. In this process, we removed the degraded corner points but did not remove excessive local features to ensure the authenticity of the bone morphology. After fitting the surface, we performed a deviation analysis and further adjusted the polygon shape based on the results. Models of cortical and cancellous bone were developed using Boolean operations, and models of the proximal femoral bone were built in an assembling pattern.

### Modelling of fractures and internal fixation models

**Fig. 1:**
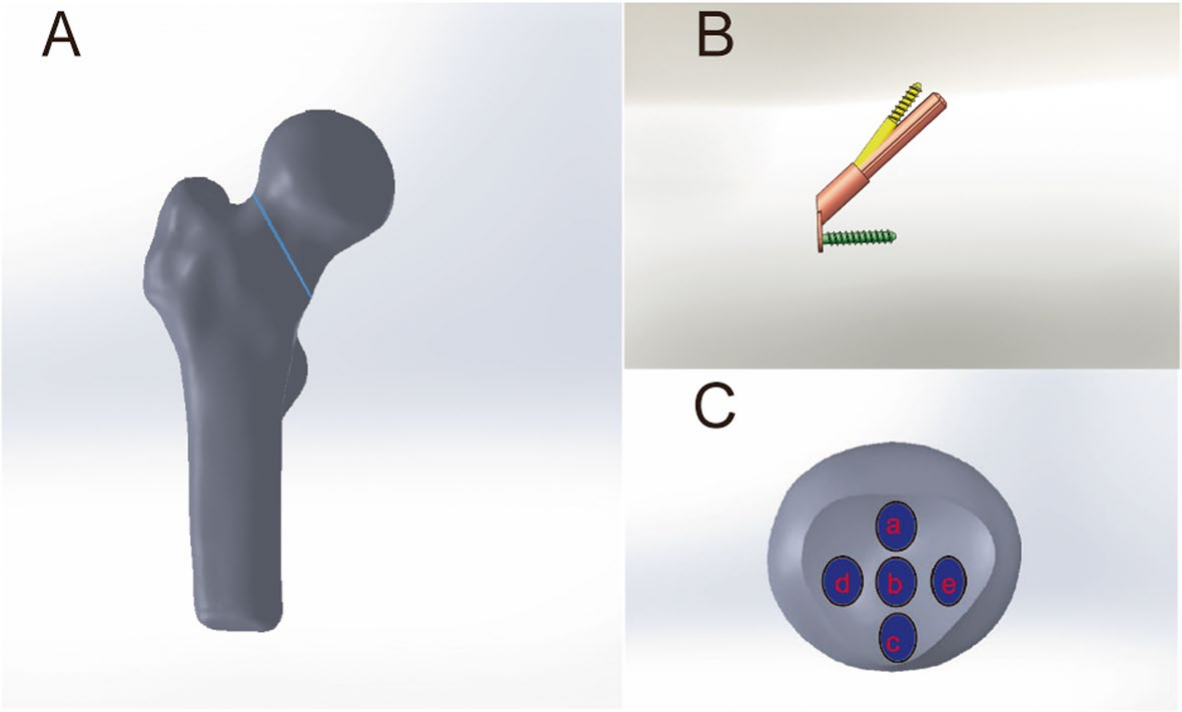
Pauwels type III fracture model **A**; FNS model **B**; Sagittal positions of bolt **C**: superior(a), central(b), inferior(c), anterior(d), posterior(e)

In order to develop a Pauwels type III fracture model, the fracture line was cut, and the Pauwels angle was set at 60 (Fig. 1A). The schematic drawing function was used to reconstruct models of internal fixation based on the present clinical characteristics (DePuy Synthes, Zuchwil, Switzerland). (Fig. 1B). We selected a 1-hole steel plate with bolts of 5 mm increments. FNS was placed in five locations (superior, central, inferior, anterior, posterior) in the femoral neck (Fig. 1C). This distance remained the same between the subchondral bone and the main nail tip (TAD < 25 mm). After all, parts had been detected by interference in order to evaluate their process quality, they were imported into Ansys 17.0 for further analysis.

### Parameter setting and meshing

**Fig. 2:**
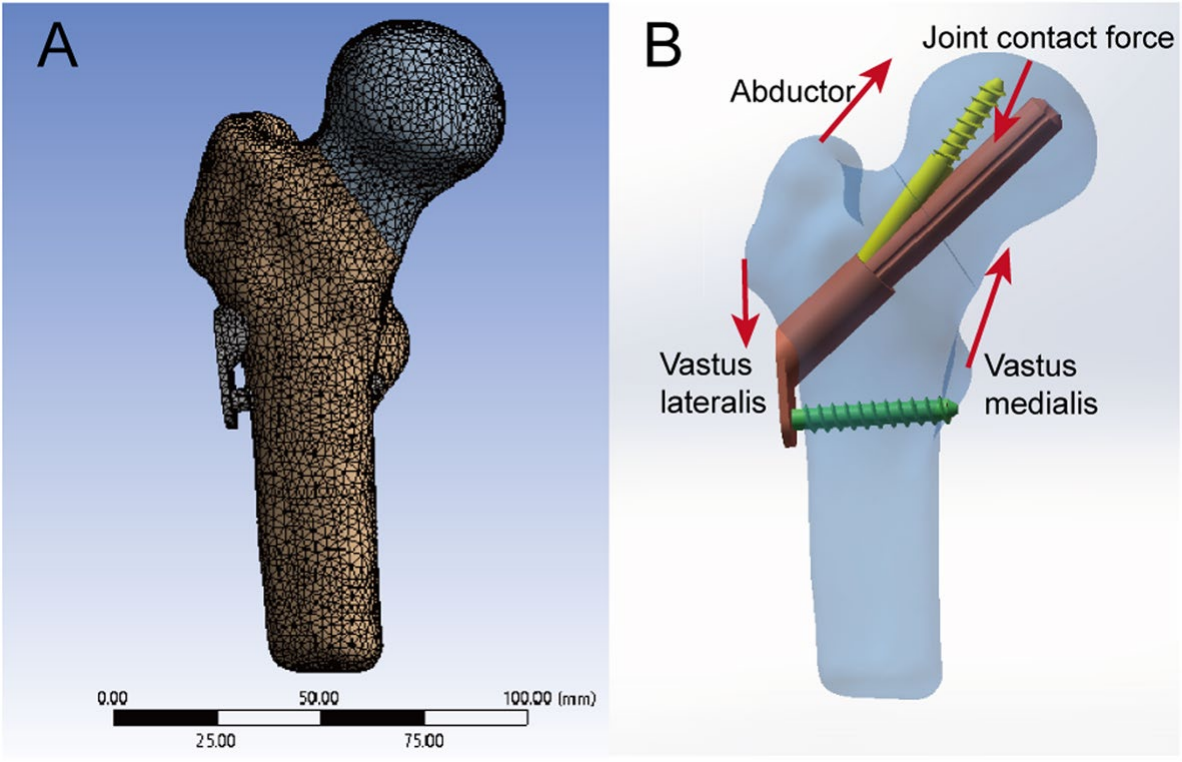
Tetrahedral mesh schematic **A**, Mechanical model of the hip joint muscles **B**

A homogeneous and isotropic environment was created for the implants and bone in this study [14]. The model including cortical bone, cancellous bone, and internal fixations was assigned to different elastic moduli and Poisson's ratios in accordance with the previously specified material property parameters [15, 16]. (Table 1) The mesh size was selected in accordance with the convergence experiment. Since the geometry of nail was complex, the mesh consists primarily of tetrahedral elements (Fig. 2A).

**Table 1:** Material properties of models in this study

Item	Elastic modulus(MPa)	Poisson's ratio
Femoral cancellous bone	840	0. 2
Femoral cortical bone	16,800	0. 3
Femoral Neck System	105,000	0. 35

### Boundary conditions and loads

The connection between the fracture surface was set to friction, and the coefficient of friction was 0.46 [17]. The relationship between the internal fixations and bone was set as binding relation.

The distal femur's degrees of freedom were set to zero in six directions. In view of the angle between the coronal and sagittal surfaces of the femoral neck, the reaction force of the joint surface is vector force. The load vector of 1800 N corresponds to 300% for a body weight of 60 kg kg. Based on Lin's [18] research, the mechanical model of the hip joint muscles has been developed (Fig. 2B). In accordance with the convergence study, no special errors were observed during the execution of the solution.

### Evaluation indicators

The same stress load was applied to the five models in the static analysis mode. Equivalent stress, shear stress, and total deformation were added to resultant analysis. The peak value of internal fixation and fracture surface was the most relevant in the documentation of equivalent forces. The total deformation of the model and the internal fixed shear stress were recorded. Meanwhile, the distribution of equivalent force and shear stress was also shown in the graph.

## Results

**Fig. 3:**
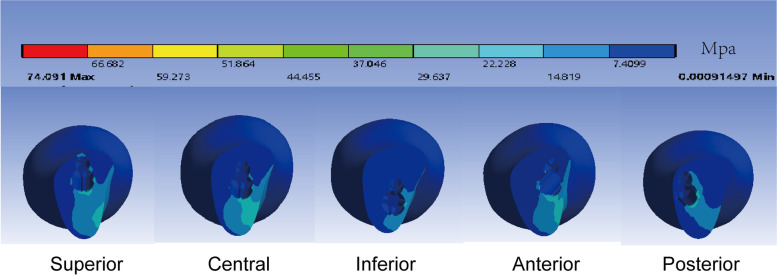
Distribution of interfragmentary stress

Figure 3 illustrates the interfragmentary stress distribution. Among the fracture surfaces, stress was mainly concentrated around the screws and in the medial femoral neck. The interfragmentary stress was 19.02 Mpa, 18.25 Mpa, 21.12 Mpa, 20.71 Mpa, and25.80 Mpa in superior, central, inferior, anterior, and posterior models respectively. As the distance between the internal fixation and the medial cortex increased, so did the area of force on the bone.

**Fig. 4:**
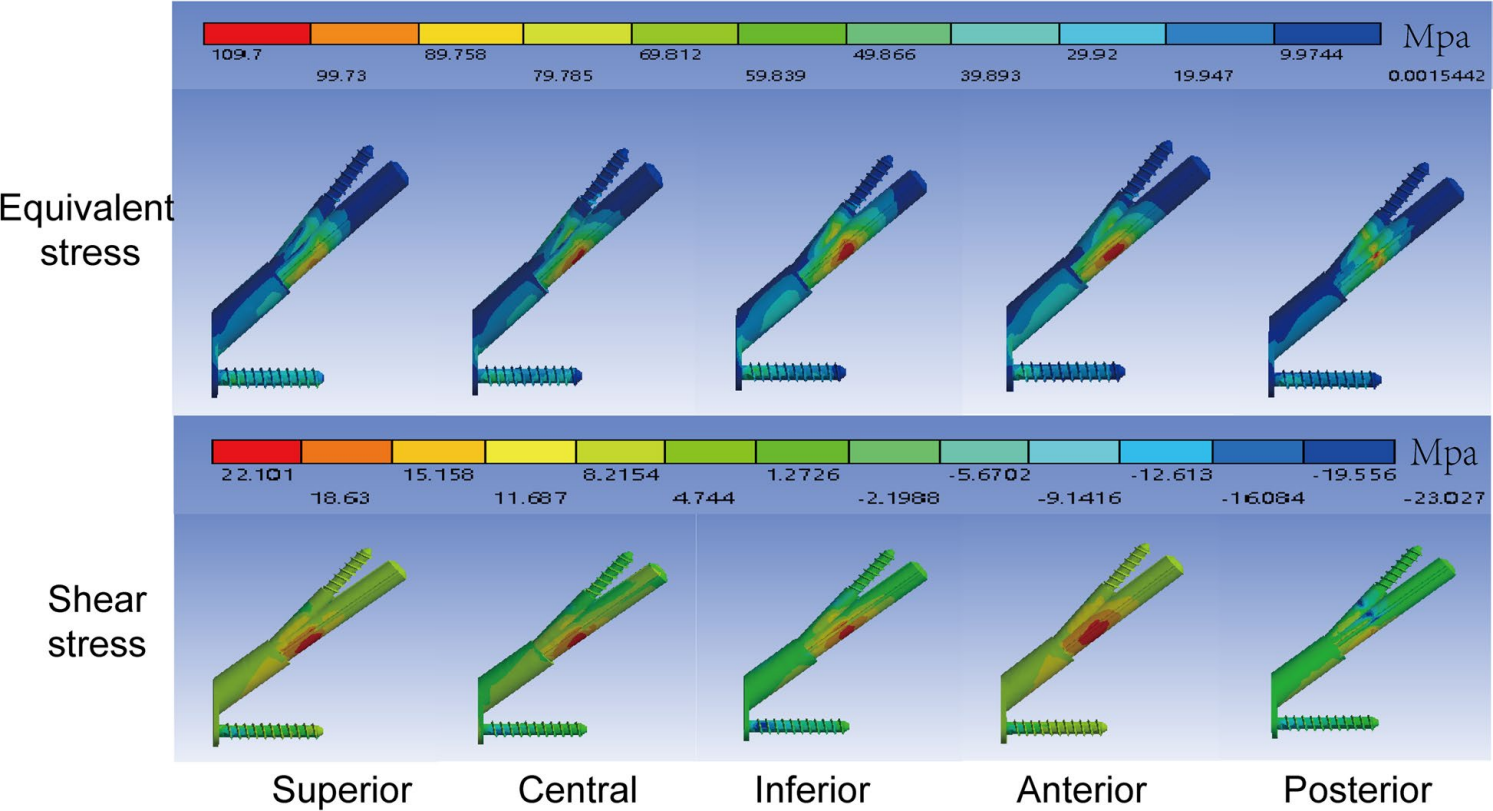
Distribution of equivalent stress and shear stress. Contour cloud map shown as coloured bands. Darker colours indicate higher stress values

The distribution of equivalent stress and shear force was displayed in Fig. 4. Shear stress primarily occurred at the contact between the fracture surface and the internal fixation. The equivalent stress was 70.9 Mpa, 74.09 Mpa, 89.27 Mpa, 75.65 Mpa, and 109.7 Mpa in superior, central, inferior, anterior, and posterior models respectively. The shear stress of internal fixation was 13.06 Mpa, 17.53 Mpa, 21.12 Mpa, 18.54 Mpa, and 22.11 Mpa in superior, central, inferior, anterior, and posterior model respectively. Total displacements were 1.399 mm, 1.276 mm, 1.345 mm, 1.306 mm and 1.336 mm for superior, central, inferior, anterior, and posterior models respectively.

**Fig. 5:**
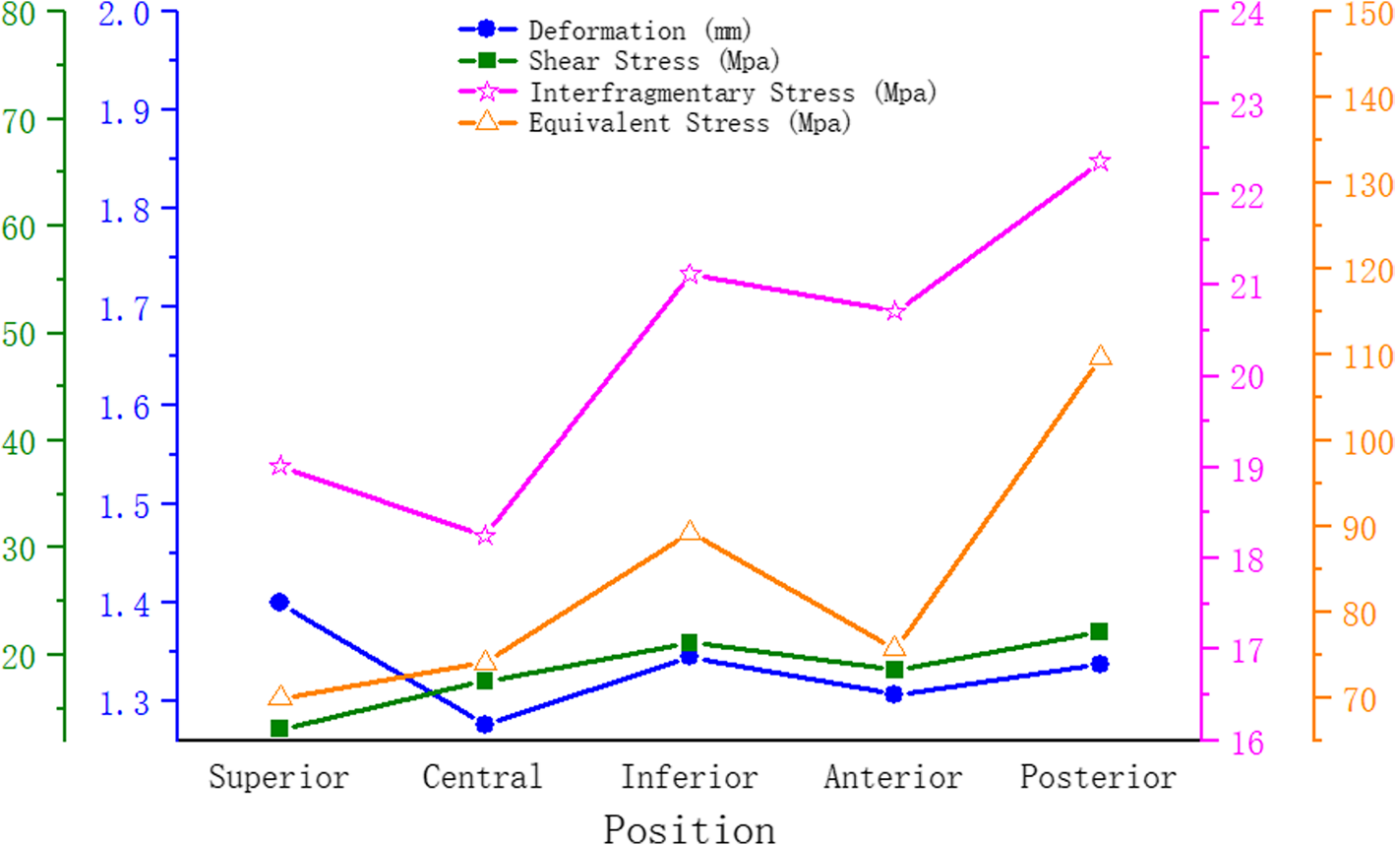
Peak point line diagram of equivalent stress, shear stress, and total deformation

The trend of equivalent stress, shear stress, and total deformation is described in Fig. 5. According to the results, the central model exhibited the smallest stress and displacement, with the exception of the superior model. A comparison of the superior model with the central model revealed that equivalent stress and shear force in the upper model were low, but the displacement and interfragmentary stress were high. This indicated that more stress was transferred to the bone near the fracture, resulting in increased displacement of the model. In general, the total displacement is positively related to the overall stability of the model. In all models, the largest stress area appeared in the superior model.

## Discussion

Clinical studies have demonstrated that FNS is a safe and effective strategy for internal fixation [19, 20]. Although manufacturers provide relatively clear steps and standards for operation, application in actual clinical practice is inconsistent. According to the clinical feedback, in order to ensure that the locking nail is located in the medullary cavity of the bone stem, it is recommended to confirm the position of the anti-spin nail before implanting the anti-spin nail. This may be due to individual patient differences and the proficiency of the physician. Meanwhile, many scholars have optimized the application of FNS. For increased stability, Fan [17] suggested using a two-hole FNS when the fracture line angle exceeded 70°. Jung [13] studied the surgical variations of using FNS to stabilize Pauwels type III femoral neck fractures. In his opinion, a gap between the femoral stem and the plate is an effective method of controlling the bolt length. In studies on intertrochanteric fractures, TAD (Tip-Apex Distance) was used to assess the stability of internal fixation [21]. However, the biomechanical performance of femoral neck fractures is not identical to that of intertrochanteric fractures. External forces acting on the femur are directed from the center of the femoral head to the femoral spine medial to the lesser trochanter, and then through the femoral stem. Is it necessary to improve the method of evaluating the position of the FNS in femoral neck fractures? Researchers have enhanced the technique of precisely adjusting the bolt depth for femoral neck fracture dislocations using FNS bolts manufactured in 5 mm increments [22]**.** Previous studies have shown that a position in the middle of the coronal plane may be preferable. A further investigation of similar findings in the sagittal plane is necessary.

As a result, we would like to examine the biomechanical properties of variation of FNS in the sagittal plane with the aid of finite element analysis. As opposed to traditional mechanical experiments, FEA provides the same mechanical environment. Mechanical results are influenced by the morphology of the femoral head, bone density, anterior inclination of the femoral neck, etc. Although it does not fully simulate the in vitro situation, this homogeneous setup can still improve the credibility and repeatability of the experiment. In our study, all models and working conditions were the same, and the only variable was the position in the sagittal plane. Moreover, meshing is a very selective process in finite element analysis. There is no doubt that the calculations of the hexahedron are fast, whereas the calculations of the tetrahedron are slow. Since the proximal femur is irregular, further simplification and cutting of the model are required if a hexahedral mesh is employed. In previous studies, we applied hexahedra in the analysis of laminar-like structures such as intervertebral discs, while irregular bones were analyzed using tetrahedra more. Since the bolt diameter of FNS is much larger than a normal screw, excessive refinement of sagittal positions in the femoral head is not necessary.

Equivalent stress, shear stress, and total deformation were recorded in our study. Equivalent stress cloud diagrams can help us understand the force distribution very well. It uses stress contours to represent the stress distribution within the model, which clearly depicts how a result varies throughout the model, allowing the analyst to quickly identify the most dangerous areas of the model. In the model, the role of internal fixation is to take up and distribute the stress. In other words, the lower the stress value of the screw, the lighter the color of the stressed area, the higher the stability, because excessive stress increases the fatigue of the material. In our research, equivalent stress primarily occurred at the contact between the fracture surface and the internal fixation, which was consistent with the force situation (Fig. 4). However, we found that there was no stress concentration region in the superior model, indicating that the internal fixation did not play a role in dispersing the stress. Combined with the stresses on the fracture surface, our hypothesis was further confirmed. The internal fixation stress of the superior model was 4.3% smaller than that of the central model. While, the interfragmentary stress of the superior model was 4.2% larger than that of the central model. We also observed that the superior model had the largest range of forces on the fracture surface. Generally, the larger the area under stress, the more bone deformation occurs and the less stable it is. Also, our data showed the displacement of the superior model increased by 9% compared to the central model. Clearly, the superior model's internal fixation system was ineffective.

Femoral head pressure load can be separated into compressive and shear stresses based on the central axis of the neck and fracture surface. Axial compressive stress can promote fracture healing. The presence of shear stress increases the relative slip between fracture surfaces, which can lead to the failure of the fixation model. Nonetheless, previous finite element analyses did not include shear stress results. In our study, the area of concentration of shear stress varied with the location of the internal fixation. This eccentric placement may lead to uneven forces, thus weakening the compression effect on the fracture surface. The superior placement kept the screw away from the pressure trabeculae and did not effectively support and share the pressure from the femoral head. Generally, the shear stress area reflects the eccentricity of the screw. In the anterior position, the shear resistance area was the largest, indicating the greatest eccentricity at the moment (Fig. 4). According to our hypothesis, this was due to the anteversion of the femoral neck. As the bolt was placed anteriorly, the plate was located on the posterior side of the proximal femur, resulting in a large angle between the bolt and the axis of the femoral neck. Results indicated that the superior model had the lowest shear stress, but its shear resistance area was larger than that of the central and inferior model. Combined with the shear resistance area and shear stress, the central location was more preferable.We combined all the results into a dotted line graph so that we could find patterns (Fig. 5). Based on the previous analysis, we considered the superior model to be the most unstable. And the central model exhibited better biomechanical performance in terms of equivalent stress, shear stress, and total deformation. This result was in accordance with the previous findings in the coronal plane.

Nevertheless, this study does have some limitations. First, the proximal femur rather than the entire length of the femur was used for modeling, but it could reflect the trend of change. Second, we ignored the force variation in the healing process of femoral neck fracture. Third, biomechanical analysis of stable fractures was not been studied. Whether the pattern of the present study is consistent in stable fractures requires further analysis.

## Conclusions

In conclusion, central position in the sagittal plane allowed axial compression while reducing shear stress of internal fixation and interfragmentary equivalent stress. Off-axis fixation of the femoral neck increased the strain area and total displacement of the bone, which in turn raised the risk of fixation failure. Therefore, the central placement of FNS may be a better surgical target in the treatment of femoral neck fractures.

## Data Availability

The datasets used and/or analysed during the current study are available from the corresponding author on reasonable request.

## Abbreviations

FNS: Femoral Neck System
CSS: Cannulated screws
DHS: Dynamic hip screw
FEA: Finite element analysis
TAD: Tip-Apex Distance
